# Functional annotation of human long noncoding RNAs via molecular phenotyping

**DOI:** 10.1101/gr.254219.119

**Published:** 2020-07

**Authors:** Jordan A. Ramilowski, Chi Wai Yip, Saumya Agrawal, Jen-Chien Chang, Yari Ciani, Ivan V. Kulakovskiy, Mickaël Mendez, Jasmine Li Ching Ooi, John F. Ouyang, Nick Parkinson, Andreas Petri, Leonie Roos, Jessica Severin, Kayoko Yasuzawa, Imad Abugessaisa, Altuna Akalin, Ivan V. Antonov, Erik Arner, Alessandro Bonetti, Hidemasa Bono, Beatrice Borsari, Frank Brombacher, Christopher J.F. Cameron, Carlo Vittorio Cannistraci, Ryan Cardenas, Melissa Cardon, Howard Chang, Josée Dostie, Luca Ducoli, Alexander Favorov, Alexandre Fort, Diego Garrido, Noa Gil, Juliette Gimenez, Reto Guler, Lusy Handoko, Jayson Harshbarger, Akira Hasegawa, Yuki Hasegawa, Kosuke Hashimoto, Norihito Hayatsu, Peter Heutink, Tetsuro Hirose, Eddie L. Imada, Masayoshi Itoh, Bogumil Kaczkowski, Aditi Kanhere, Emily Kawabata, Hideya Kawaji, Tsugumi Kawashima, S. Thomas Kelly, Miki Kojima, Naoto Kondo, Haruhiko Koseki, Tsukasa Kouno, Anton Kratz, Mariola Kurowska-Stolarska, Andrew Tae Jun Kwon, Jeffrey Leek, Andreas Lennartsson, Marina Lizio, Fernando López-Redondo, Joachim Luginbühl, Shiori Maeda, Vsevolod J. Makeev, Luigi Marchionni, Yulia A. Medvedeva, Aki Minoda, Ferenc Müller, Manuel Muñoz-Aguirre, Mitsuyoshi Murata, Hiromi Nishiyori, Kazuhiro R. Nitta, Shuhei Noguchi, Yukihiko Noro, Ramil Nurtdinov, Yasushi Okazaki, Valerio Orlando, Denis Paquette, Callum J.C. Parr, Owen J.L. Rackham, Patrizia Rizzu, Diego Fernando Sánchez Martinez, Albin Sandelin, Pillay Sanjana, Colin A.M. Semple, Youtaro Shibayama, Divya M. Sivaraman, Takahiro Suzuki, Suzannah C. Szumowski, Michihira Tagami, Martin S. Taylor, Chikashi Terao, Malte Thodberg, Supat Thongjuea, Vidisha Tripathi, Igor Ulitsky, Roberto Verardo, Ilya E. Vorontsov, Chinatsu Yamamoto, Robert S. Young, J. Kenneth Baillie, Alistair R.R. Forrest, Roderic Guigó, Michael M. Hoffman, Chung Chau Hon, Takeya Kasukawa, Sakari Kauppinen, Juha Kere, Boris Lenhard, Claudio Schneider, Harukazu Suzuki, Ken Yagi, Michiel J.L. de Hoon, Jay W. Shin, Piero Carninci

**Affiliations:** 1 RIKEN Center for Integrative Medical Sciences, Yokohama, Kanagawa 230-0045, Japan;; 2 RIKEN Center for Life Science Technologies, Yokohama, Kanagawa 230-0045, Japan;; 3 Laboratorio Nazionale Consorzio Interuniversitario Biotecnologie (CIB), Trieste 34127, Italy;; 4 Engelhardt Institute of Molecular Biology, Russian Academy of Sciences, Moscow 119991, Russia;; 5 Institute of Protein Research, Russian Academy of Sciences, Pushchino 142290, Russia;; 6 Department of Computer Science, University of Toronto, Toronto, Ontario M5S 1A1, Canada;; 7 Program in Cardiovascular and Metabolic Disorders, Duke-National University of Singapore Medical School, Singapore 169857, Singapore;; 8 Roslin Institute, University of Edinburgh, Edinburgh EH25 9RG, United Kingdom;; 9 Center for RNA Medicine, Department of Clinical Medicine, Aalborg University, Copenhagen 9220, Denmark;; 10 Institute of Clinical Sciences, Faculty of Medicine, Imperial College London, London W12 0NN, United Kingdom;; 11 Computational Regulatory Genomics, MRC London Institute of Medical Sciences, London W12 0NN, United Kingdom;; 12 Berlin Institute for Medical Systems Biology, Max Delbrük Center for Molecular Medicine in the Helmholtz Association, Berlin 13125, Germany;; 13 Institute of Bioengineering, Research Center of Biotechnology, Russian Academy of Sciences, Moscow 117312, Russia;; 14 Graduate School of Integrated Sciences for Life, Hiroshima University, Higashi-Hiroshima City 739-0046, Japan;; 15 Centre for Genomic Regulation (CRG), The Barcelona Institute of Science and Technology, Barcelona, Catalonia 08003, Spain;; 16 International Centre for Genetic Engineering and Biotechnology (ICGEB), University of Cape Town, Cape Town 7925, South Africa;; 17 Institute of Infectious Diseases and Molecular Medicine (IDM), Department of Pathology, Division of Immunology and South African Medical Research Council (SAMRC) Immunology of Infectious Diseases, Faculty of Health Sciences, University of Cape Town, Cape Town 7925, South Africa;; 18 School of Computer Science, McGill University, Montréal, Québec H3G 1Y6, Canada;; 19 Biomedical Cybernetics Group, Biotechnology Center (BIOTEC), Center for Molecular and Cellular Bioengineering (CMCB), Center for Systems Biology Dresden (CSBD), Cluster of Excellence Physics of Life (PoL), Department of Physics, Technische Universität Dresden, Dresden 01062, Germany;; 20 Center for Complex Network Intelligence (CCNI) at the Tsinghua Laboratory of Brain and Intelligence (THBI), Department of Bioengineering, Tsinghua University, Beijing 100084, China;; 21 Institute of Cancer and Genomic Sciences, College of Medical and Dental Sciences, University of Birmingham, Birmingham B15 2TT, United Kingdom;; 22 Center for Personal Dynamic Regulome, Stanford University, Stanford, California 94305, USA;; 23 Department of Biochemistry, Rosalind and Morris Goodman Cancer Research Center, McGill University, Montréal, Québec H3G 1Y6, Canada;; 24 Institute of Pharmaceutical Sciences, Swiss Federal Institute of Technology, Zurich 8093, Switzerland;; 25 Department of Computational Systems Biology, Vavilov Institute of General Genetics, Russian Academy of Sciences, Moscow 119991, Russia;; 26 Department of Oncology, Johns Hopkins University, Baltimore, Maryland 21287, USA;; 27 Department of Biological Regulation, Weizmann Institute of Science, Rehovot 76100, Israel;; 28 Epigenetics and Genome Reprogramming Laboratory, IRCCS Fondazione Santa Lucia, Rome 00179, Italy;; 29 Genome Biology of Neurodegenerative Diseases, German Center for Neurodegenerative Diseases (DZNE), Tübingen 72076, Germany;; 30 Graduate School of Frontier Biosciences, Osaka University, Suita 565-0871, Japan;; 31 RIKEN Preventive Medicine and Diagnosis Innovation Program (PMI), Saitama 351-0198, Japan;; 32 Institute of Infection, Immunity, and Inflammation, University of Glasgow, Glasgow, Scotland G12 8QQ, United Kingdom;; 33 Department of Biosciences and Nutrition, Karolinska Institutet, Huddinge 14157, Sweden;; 34 Moscow Institute of Physics and Technology, Dolgoprudny 141701, Russia;; 35 Biological and Environmental Sciences and Engineering Division, King Abdullah University of Science and Technology, Thuwal 23955-6900, Kingdom of Saudi Arabia;; 36 Department of Biology and BRIC, University of Copenhagen, Denmark, Copenhagen N DK2200, Denmark;; 37 MRC Human Genetics Unit, University of Edinburgh, Edinburgh EH4 2XU, United Kingdom;; 38 National Centre for Cell Science, Pune, Maharashtra 411007, India;; 39 Centre for Global Health Research, Usher Institute, University of Edinburgh, Edinburgh EH8 9AG, United Kingdom;; 40 Harry Perkins Institute of Medical Research, QEII Medical Centre and Centre for Medical Research, The University of Western Australia, Nedlands, Perth, Western Australia 6009, Australia;; 41 Universitat Pompeu Fabra (UPF), Barcelona, Catalonia 08002, Spain;; 42 Princess Margaret Cancer Centre, Toronto, Ontario M5G 1L7, Canada;; 43 Stem Cells and Metabolism Research Program, University of Helsinki and Folkhälsan Research Center, 00290 Helsinki, Finland;; 44 Sars International Centre for Marine Molecular Biology, University of Bergen, Bergen N-5008, Norway;; 45 Department of Medicine and Consorzio Interuniversitario Biotecnologie p.zle Kolbe 1 University of Udine, Udine 33100, Italy;; 46 Department of Molecular Biophysics and Biochemistry, Yale University, New Haven, Connecticut 06510, USA

## Abstract

Long noncoding RNAs (lncRNAs) constitute the majority of transcripts in the mammalian genomes, and yet, their functions remain largely unknown. As part of the FANTOM6 project, we systematically knocked down the expression of 285 lncRNAs in human dermal fibroblasts and quantified cellular growth, morphological changes, and transcriptomic responses using Capped Analysis of Gene Expression (CAGE). Antisense oligonucleotides targeting the same lncRNAs exhibited global concordance, and the molecular phenotype, measured by CAGE, recapitulated the observed cellular phenotypes while providing additional insights on the affected genes and pathways. Here, we disseminate the largest-to-date lncRNA knockdown data set with molecular phenotyping (over 1000 CAGE deep-sequencing libraries) for further exploration and highlight functional roles for *ZNF213-AS1* and *lnc-KHDC3L-2*.

Over 50,000 loci in the human genome transcribe long noncoding RNAs (lncRNAs) (Iyer et al. 2015; Hon et al. 2017), which are defined as transcripts at least 200 nucleotides (nt) long with low or no protein-coding potential. Although lncRNA genes outnumber protein-coding genes in mammalian genomes, they are comparatively less conserved (Ulitsky 2016), lowly expressed, and more cell-type-specific (Hon et al. 2017). However, the evolutionary conservation of lncRNA promoters (Carninci et al. 2005) and the structural motifs of lncRNAs (Chu et al. 2015; Xue et al. 2016) suggest that lncRNAs are fundamental biological regulators. To date, only a few hundred human lncRNAs have been extensively characterized (de Hoon et al. 2015; Quek et al. 2015; Volders et al. 2015; Ma et al. 2019), revealing their roles in regulating transcription (Engreitz et al. 2016b), translation (Carrieri et al. 2012), and chromatin state (Gupta et al. 2010; Guttman et al. 2011; Guttman and Rinn 2012; Quinn and Chang 2016; Ransohoff et al. 2018).

Our recent FANTOM5 computational analysis showed that 19,175 (out of 27,919) human lncRNA loci are functionally implicated (Hon et al. 2017). Yet, genomic screens are necessary to comprehensively characterize each lncRNA. One common approach of gene knockdown followed by a cellular phenotype assay typically characterizes a small percentage of lncRNAs for a single observable phenotype. For example, a recent large-scale screening using CRISPR interference (CRISPRi) found that ∼3.7% of targeted lncRNA loci are essential for cell growth or viability in a cell-type-specific manner (Liu et al. 2017). In addition, CRISPR-Cas9 experiments targeting splice sites identified ∼2.1% of lncRNAs that affect growth of K562 (Liu et al. 2018), and a CRISPR activation study revealed ∼0.11% lncRNAs to be important for drug resistance in melanoma (Joung et al. 2017). However, many of these studies target the genomic DNA, potentially perturbing the chromatin architecture, or focus on a single cellular assay, possibly missing other relevant functions and underlying molecular pathways.

As a part of the FANTOM6 pilot project, we established an automated high-throughput cell culture platform to suppress 285 lncRNAs expressed in human primary dermal fibroblasts (HDFs) using antisense LNA-modified GapmeR antisense oligonucleotide (ASO) technology (Roux et al. 2017). We then quantified the effect of each knockdown on cell growth and morphology using real-time imaging, followed by Cap Analysis Gene Expression (CAGE) (Murata et al. 2014) deep sequencing to reveal molecular pathways associated with each lncRNA. In contrast to cellular phenotyping, molecular phenotyping provides a detailed assessment of the response to a lncRNA knockdown at the molecular level, allowing biological pathways to be associated to lncRNAs even in the absence of an observable cellular phenotype. All data and analysis results are publicly available (see Data access), and results can be interactively explored using our in-house portal (https://fantom.gsc.riken.jp/zenbu/reports/#FANTOM6).

## Results

### Selection and ASO-mediated knockdown of lncRNA targets

**Figure 1.**
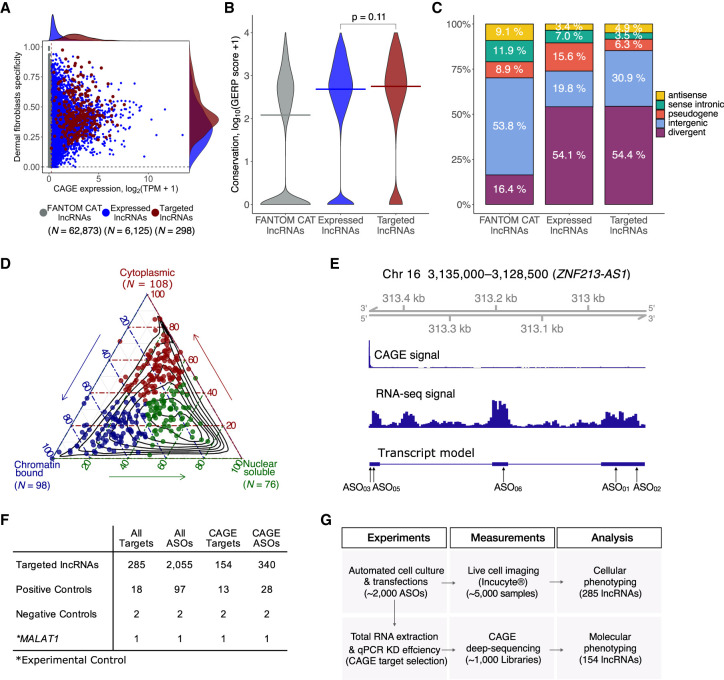
Selection of lncRNA targets, their properties, and the study overview. (*A*) CAGE expression levels at log_2_TPM (tags per million) and human dermal fibroblasts (HDFs) specificity of lncRNAs in the FANTOM CAT catalog (Hon et al. 2017) (*N* = 62,873; gray), lncRNAs expressed in HDFs (*N* = 6125; blue), and targeted lncRNAs (*N* = 285; red). The dashed vertical line indicates most lowly expressed lncRNA target (∼0.2 TPM). (*B*) Gene conservation levels of lncRNAs in the FANTOM CAT catalog (gray), lncRNAs expressed in HDFs (blue), and targeted lncRNAs (red). Crossbars indicate the median. No significant difference is observed when comparing targeted and expressed in HDF lncRNAs (Wilcoxon *P* = 0.11). (*C*) Similar to that in *B* but for genomic classes of lncRNAs. Most of the targeted lncRNAs and those expressed in HDFs are expressed from divergent promoters. (*D*) Subcellular localization (based on relative abundances from RNA-seq fractionation data) for targeted lncRNAs. Chromatin-bound (*N* = 98; blue); nuclear soluble (*N* = 76; green); cytoplasmic (*N* = 108; red). Black contours represent the distribution of all lncRNAs expressed in HDFs. (*E*) Example of *ZNF213-AS1* loci showing transcript model, CAGE, and RNA-seq signal along with targeting ASOs. (*F*) Number of ASOs for target lncRNAs and controls used in the experiment. (*G*) Schematics of the study.

Human dermal fibroblasts are nontransformed primary cells that are commonly used for investigating cellular reprogramming (Takahashi et al. 2007; Ambasudhan et al. 2011), wound healing (Li and Wang 2011), fibrosis (Kendall and Feghali-Bostwick 2014), and cancer (Kalluri 2016). Here, an unbiased selection of lncRNAs expressed in HDFs was performed to choose 285 lncRNAs for functional interrogation (Methods; Supplemental Table S1; Fig. 1A–C). Using RNA-seq profiling of fractionated RNA, we annotated the lncRNA subcellular localization as the chromatin-bound (35%), nucleus-soluble (27%), or cytoplasmic (38%) (Fig. 1D). We then designed a minimum of five non-overlapping antisense oligonucleotides against each lncRNA (Supplemental Methods; Supplemental Table S2; Fig. 1E,F) and transfected them individually using an automated cell culture platform to minimize experimental variability (Fig. 1G). The overall knockdown efficiencies across 2021 ASOs resulted in median value of 45.4%, and we could successfully knockdown 879 out of 2021 (43.5%) ASOs (>40% knockdown efficiency in at least two primer pairs or >60% in one primer pair) (Supplemental Table S2). ASOs targeting exons or introns were equally effective, and knockdown efficiencies were independent of the genomic class, expression level, and subcellular localization of the lncRNA (Supplemental Fig. S1A–D).

### A subset of lncRNAs are associated with cell growth and morphology changes

**Figure 2.**
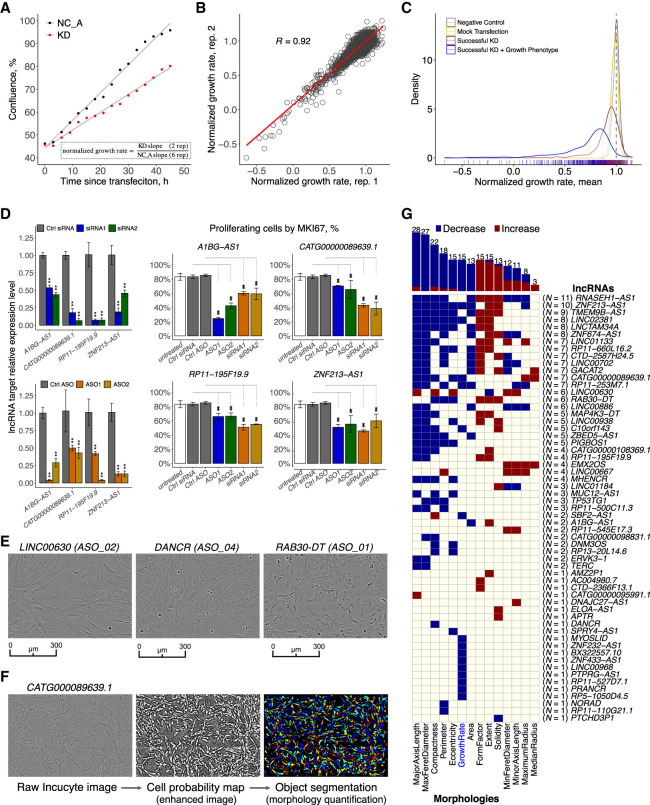
Cell growth and morphology assessment. (*A*) Selected example (*PTPRG1-AS1*) showing the normalized growth rate estimation using a matching NC_A (negative control). (*B*) Correlation of the normalized growth rate for technical duplicates across 2456 Incucyte samples. (*C*) Density distribution of normalized growth rates (technical replicates averaged) 252 ASOs targeting lncRNAs with successful knockdown (KD) and growth phenotype (blue) consistent in two replicates (FDR < 0.05 as compared to matching NC_A; 246 ASOs inhibited growth), 627 ASOs targeting lncRNAs with successful KD (purple), 270 negative control (NC_A) samples (gray), and 90 mock-transfected cells (Lipofectamine only) samples (yellow). (*D*) MKI67 staining (growth inhibition validation) for four selected lncRNA targets after siRNA and ASOs suppression. (*E*) Incucyte cell images of selected distinct cell morphologies changes upon an lncRNA KD. (*F*) An overview of the cell morphology imaging processing pipeline using a novel lncRNA target, *CATG000089639.1*, as an example. (*G*) lncRNAs (*N* = 59) significantly (FDR < 0.05) and consistently (after adjusting for the number of successfully targeting ASOs) affecting cell growth (*N* = 15) and cell morphologies (*N* = 44).

To evaluate the effect of each lncRNA knockdown on cell growth and morphology, we imaged ASO-transfected HDFs in duplicate every 3 h for a total of 48 h (Supplemental Table S3) and estimated their growth rate based on cell confluence measurements (Fig. 2A,B). First, we observed across all ASOs that changes in cell growth and morphological parameters were significantly correlated with knockdown efficiency (Supplemental Fig. S1E). Considering both successful knockdown and significant growth inhibition (Student's two-sided *t*-test FDR ≤ 0.05), 246 out of 879 ASOs (∼28%) showed cellular phenotype (Fig. 2C; Supplemental Table S3).

To assess globally whether the observed growth inhibition is lncRNA-specific, we used all 194 lncRNAs successfully targeted by at least two ASOs (Supplemental Fig. S2A) and found that ASOs targeting the same lncRNA were significantly more likely to have a concordant growth response than ASOs targeting different lncRNA (empirical *P* = 0.00037) (Supplemental Methods; Supplemental Fig. S2B). However, different ASOs targeting the same lncRNA typically showed different effects on growth, possibly due to variable knockdown efficiencies or differences in targeted lncRNA isoforms, as well as off-target effects. To reliably identify target-specific cellular phenotype, we applied conditional cutoffs based on the number of successful ASOs per each lncRNA (Supplemental Methods; Supplemental Fig. S2C) and identified 15/194 lncRNAs (7.7%) with growth phenotype (adjusted background <5%) (Supplemental Fig. S2D). We validated *A1BG-AS1*, which was previously implicated in cell growth (Bai et al. 2019), *CATG00000089639*, *RP11-195F19.9*, and *ZNF213-AS1* by measuring the MKI67 proliferation protein marker upon knockdown with siRNAs and with selected ASOs (Fig. 2D; Supplemental Fig. S2E).

In addition to cell growth, we also explored changes in cell morphology (Fig. 2E). Using a machine learning-assisted workflow (Methods), each cell was segmented and its morphological features representing various aspects of cell shapes and sizes were quantified (Fig. 2F; Supplemental Table S3; Carpenter et al. 2006). As an example, knockdown of 14/194 lncRNAs (7.2%) affected the spindle-like morphology of fibroblasts, as indicated by a consistent decrease in their observed eccentricity without reducing the cell number, suggesting possible cellular transformation toward epithelial-like states. Collectively, we observed 59/194 lncRNAs (∼30%) affecting cell growth and/or morphological parameters (Fig. 2G; Supplemental Table S3).

### Molecular phenotyping by CAGE recapitulates cellular phenotypes and highlights functions of lncRNAs

**Figure 3.**
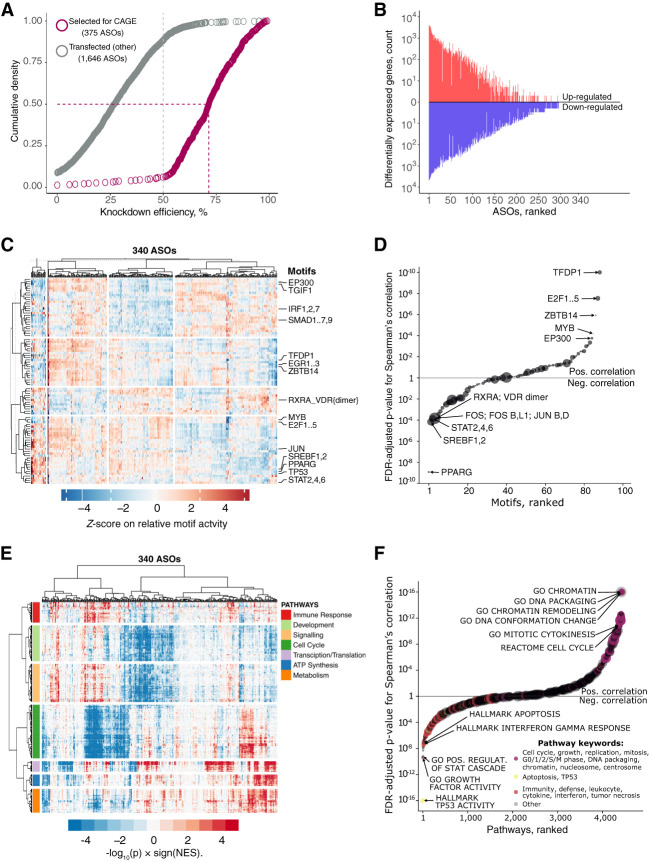
CAGE predicts cellular phenotypes. (*A*) RT-qPCR knockdown efficiency for 2021 ASO-transfected samples (targeted lncRNAs only). Gray dashed line indicates 50% KD efficiency generally required for CAGE selection. Purple dashed lines indicate median KD efficiency (71.5%) for 375 ASOs selected for CAGE sequencing. After quality control, 340 ASOs targeting lncRNAs were included for further analysis. (*B*) Distribution of significantly differentially expressed genes (up-regulated: FDR < 0.05, *Z*-score > 1.645, log_2_FC > 0.5; and down-regulated: FDR < 0.05, *Z*-score < −1.645, log_2_FC < −0.5) across all 340 ASOs. (*C*) Motif Response Activity Analysis (MARA) across 340 ASOs. Scale indicates *Z*-score of the relative motif activity (the range was set to abs[*Z*-score] = <5 for visualization purposes). (*D*) Correlation between normalized growth rate and motif activities across 340 ASOs targeting lncRNAs with highlighted examples. Motif sizes shown are scaled based on the HDF expression of their associated TFs (range 1 to ∼600 TPM). (*E*) Enriched biological pathways across 340 ASOs. Scale indicates GSEA enrichment value calculated as −log_10_(p) × sign(NES). (*F*) Same as in *D* but for selected GSEA pathways. Pathways sizes are scaled based on the number of associated genes.

Next, we selected 340 ASOs with high knockdown efficiencies (mostly >50%; median 71.4%) and sequenced 970 CAGE libraries to analyze 154 lncRNAs (Fig. 3A; Supplemental Table S4). To assess functional implications by individual ASOs, we performed differential gene expression, Motif Activity Response Analysis (MARA) (The FANTOM Consortium et al. 2009), and Gene Set Enrichment Analysis (GSEA) (Fig. 3B–F; Subramanian et al. 2005), and compared them with cellular phenotype.

We globally observed significant knockdown-mediated transcriptomic changes (which generally correlated with KD efficiency) (Supplemental Fig. S3A), with ∼57% of ASOs showing at least 10 differentially expressed genes (FDR ≤ 0.05; abs[log_2_FC] > 0.5). For 84 divergent-antisense lncRNAs (targeted by 186 independent ASOs) (Supplemental Methods), we found their partner gene to be generally unchanged (median abs[log_2_FC] = ∼0.13), with an exception of two significantly down-regulated and three significantly up-regulated genes (FDR ≤ 0.05) (Supplemental Fig. S3B). We have, however, noticed a common response in a large number of ASOs (∼30%–35% of all responding ASOs), such as down-regulation of cell-cycle-related pathways, up-regulated stress genes and pathways, or altered cell metabolism and energetics (Supplemental Fig. S3C,D).

When comparing knockdown-mediated molecular and cellular response, we found that transcription factor motifs that promote cell growth, including TFDP1, E2F1,2,3, and EP300, were positively correlated with the measured cell growth rate, whereas transcription factor motifs known to inhibit growth or induce apoptosis (e.g., PPARG, SREBPF, and STAT2,4,6) were negatively correlated (Fig. 3D; Supplemental Fig. S4A; Supplemental Table S6). Moreover, correlations of growth with GSEA pathways (Fig. 3F; Supplemental Fig. S4B; Supplemental Table S6) or with FANTOM5 coexpression clusters (Supplemental Fig. S4C) showed that cell growth and replication-related pathways were positively correlated with the measured growth rate, whereas those related to immunity, and cell stress and cell death were negatively correlated. We found that among 53 ASOs implicated in a growth-inhibition pathway based on the CAGE profiles, only 43% of them showed growth inhibition in the real-time imaging. This might suggest better sensitivity of transcriptomic profiling when detecting phenotypes as compared to live cell imaging methods, which are more prone to a delayed cellular response to the knockdown.

Additionally, morphological changes were reflected in the molecular phenotype assessed by CAGE (Supplemental Fig. S4D). Cell radius and axis length were associated with GSEA categories related to actin arrangement and cilia, whereas cell compactness was negatively correlated with apoptosis. The extensive molecular phenotyping analysis also revealed pathways not explicitly associated with cell growth and cell morphology, such as transcription, translation, metabolism, development, and signaling (Fig. 3E).

Next, to globally assess whether individual ASO knockdowns lead to lncRNA-specific effects, we scaled the expression change of each gene across the whole experiment and compared differentially expressed genes (Fig. 3B) of all possible ASO pairs targeting the same lncRNA target versus different lncRNAs (Supplemental Methods; Supplemental Table S5). We found that the concordance of the same target group was significantly greater than that of the different target group (comparing the Jaccard indices across 10,000 permutations) (Supplemental Fig. S5A), suggesting that ASO knockdowns are nonrandom and lead to more lncRNA specific effects than the nontargeting ASO pairs. Further, by requiring at least five common DEGs (FDR ≤ 0.05, abs[log_2_FC] > 0.5, abs[*Z*-score] > 1.645) and ASO-pairs significantly above the nontargeting ASO pairs background (*P* ≤ 0.05), we identified 16 ASO pairs, targeting 13 lncRNAs, exhibiting reproducible knockdown-mediated molecular responses in human dermal fibroblasts (Supplemental Fig. S5B). Corresponding GSEA pathways and MARA motifs of these 16 ASO pairs are shown in Supplemental Figure S5C.

### siRNA validation experiments

To evaluate whether the lncRNA-specific effects can be measured by other knockdown technologies, nine lncRNAs, with relatively mild growth phenotype, were subjected to siRNA knockdown. Measuring transcriptional response, we noted that higher concordance was observed for ASO modality alone (Supplemental Fig. S5D). The observed discrepancies in the transcriptional response between ASO- and siRNA-mediated knockdowns could be contributed by their mode of action and variable activities in different subcellular compartments. Next, a concordant response was found for (5/36) ASO-siRNA pairs targeting three lncRNAs (Supplemental Fig. S5E; Supplemental Table S5), enriched in the cytoplasm (*MAPKAPK5-AS1*), soluble nuclear fraction (*LINC02454*), and in the chromatin-bound fraction (*A1BG-AS1*). Although we cannot completely exclude the technical artifacts of each technology, concordant cellular response exhibited by using ASOs alone suggests that lncRNAs, in part, are essential regulatory elements in cells. Yet, our study generally warrants a careful assessment of specific findings from different knockdown technologies, including CRISPR-inhibition, and demonstrates a requirement of using multiple replicates in a given target per each modality.

### *ZNF213-AS1* is associated with cell growth and migration

**Figure 4.**
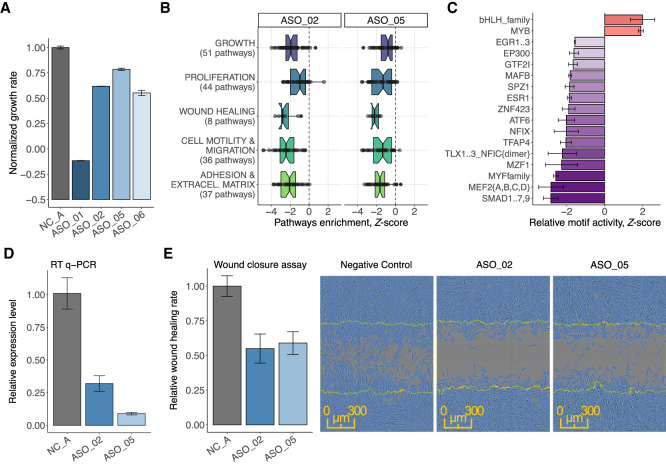
*ZNF213-AS1* regulates cell growth, migration, and proliferation. (*A*) Normalized growth rate across four distinct ASOs (in duplicate) targeting *ZNF213-AS1* as compared to six negative control samples (shown in gray). (*B*) Enrichment of biological pathways associated with growth, proliferation, wound healing, migration, and adhesion for ASO_02 and ASO_05. (*C*) Most consistently down- and up-regulated transcription factor binding motifs including those for transcription factors known to modulate growth, migration, and proliferation such as for example EGR family, EP300, GTF2I. (*D*) Knockdown efficiency measured by RT-qPCR after wound closure assay (72 h posttransfection) showing sustained suppression (65%–90%) of *ZNF213-AS1*. (*E*) Transfected, replated, and mitomycin C (5 µg/mL)-treated HDF cells were scratched and monitored in the Incucyte imaging system. Relative wound closure rate calculated during the 24 h postscratching shows 40%–45% reduction for the two targeting ASOs (ASO_02 [*N* = 10] and ASO_05 [*N* = 13]) as compared to NC_A transfection controls (*N* = 33, shown in gray) and the representative images of wound closure assay 16 h postscratching.

Extensive molecular and cellular phenotype data for each ASO knockdown can be explored using our portal https://fantom.gsc.riken.jp/zenbu/reports/#FANTOM6. As an example of an lncRNA associated with cell growth and morphology (Fig. 2G), we showcase *ZNF213-AS1* (*RP11-473M20.14*). This lncRNA is highly conserved in placental mammals, moderately expressed (∼eight CAGE tags per million) in HDFs, and enriched in the chromatin-bound fraction. Four distinct ASOs (ASO_01, ASO_02, ASO_05, and ASO_06) strongly suppressed expression of *ZNF213-AS1*, whereas expression of the *ZNF213* sense gene was not significantly affected in any of the knockdowns. The four ASOs caused varying degrees of cell growth inhibition (Fig. 4A). ASO_01 and ASO_06 showed a reduction in cell number, as well as an up-regulation of apoptosis and immune and defense pathways in GSEA, suggesting cell death. While cell growth inhibition observed for ASO_02 and ASO_05 was confirmed by MKI67 marker staining (Fig. 2D; Supplemental Table S7), the molecular phenotype revealed suppression of GSEA pathways related to cell growth, as well as to cell proliferation, motility, and extracellular structure organization (Fig. 4B). We also observed consistent down-regulation of motifs related to the observed cellular phenotype, for example, EGR1, EP300, SMAD1…7,9 (Fig. 4C).

As cell motility pathways were affected by the knockdown, we tested whether *ZNF213-AS1* could influence cell migration. Based on the wound-closure assay after transient cell growth inhibition (mitomycin C and serum starvation) (Supplemental Fig. S2F,G), we observed a substantial reduction of wound closure rate (∼40% over a 24-h period) in the *ZNF213-AS1-*depleted HDFs (Fig. 4D,E). The reduced wound healing rate should thus mainly reflect reduced cell motility, further confirming affected motility pathways predicted by the molecular phenotype.

As these results indicated a potential role of *ZNF213-AS1* in cell growth and migration, we used FANTOM CAT Recount 2 atlas (Imada et al. 2020), which incorporates The Cancer Genome Atlas (TCGA) data set (Collado-Torres et al. 2017), and found relatively higher expression of *ZNF213-AS1* in acute myeloid leukemia (LAML) and in low-grade gliomas (LGG) as compared to other cancers (Supplemental Fig. S6A). In LAML, the highest expression levels were associated with mostly undifferentiated states, whereas in LGG, elevated expression levels were found in oligodendrogliomas, astrocytomas, and in IDH1 mutated tumors, suggesting that *ZNF213-AS1* is involved in modulating differentiation and proliferation of tumors (Supplemental Fig. S6B–E). Further, univariate Cox proportional hazard analysis as well as Kaplan-Meier curves for LGG were significant and consistent with our findings (HR = 0.61, BH FDR = 0.0079). The same survival analysis on LAML showed a weak association with poor prognostic outcome, but the results were not significant (Supplemental Fig. S6F,G).

### *RP11-398K22.12* (*KHDC3L-2*) regulates *KCNQ5* in *cis*

**Figure 5.**
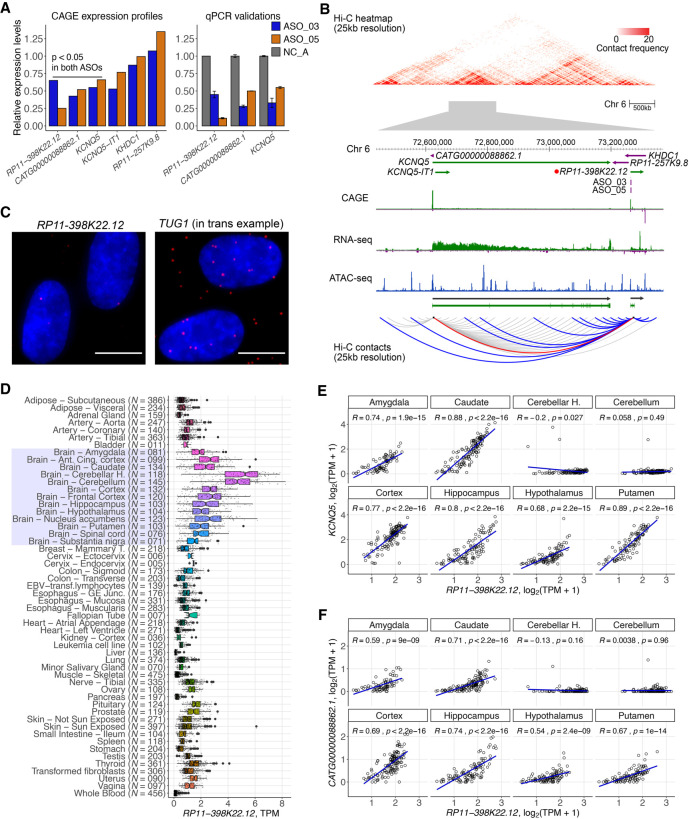
*RP11-398K22.12* down-regulates *KCNQ5* and *CATG00000088862.1* in *cis*. (*A*) Changes in expression levels of detectable genes in the same topologically associated domain (TAD) as *RP11-398K22.12* based on Hi-C analysis. Both *KCNQ5* and *CATG00000088862.1* are down-regulated (*P* < 0.05) upon the knockdown of *RP11-398K22.12* by two independent ASOs in CAGE analysis (*left*) as further confirmed with RT-qPCR (*right*). (*B*) (*Top*) Representation of the chromatin conformation in the 4-Mb region proximal to the TAD containing *RP11-398K22.12,* followed by the locus gene annotation, CAGE, RNA-seq, and ATAC-seq data for native HDFs. (*Bottom*) Schematic diagram showing Hi-C predicted contacts of *RP11-398K22.12* (blue) and *KCNQ5* (gray) (25-kb resolution, frequency ≥ 5) in HDF cells. Red line indicates *RP11-398K22.12* and *KCNQ5* contact. (*C*) FISH image for *RP11-398K22.12*, suggesting proximal regulation. *TUG1* FISH image (suggesting *trans* regulation) is included as a comparison; (bar = 10 µm). (*D*) GTEx atlas across 54 tissues (*N* = 9662 samples) shows relatively high expression levels of *RP11-398K22.12* in 13 distinct brain regions samples (highlighted). (*E*) Expression correlation for *RP11-398K22.12* and *KCNQ5* in eight out of 13 distinct brain regions, as highlighted in *D*. (*F*) Expression correlation for *RP11-398K22.12* and *CATG00000088862.1* in eight out of 13 distinct brain regions, as highlighted in *D*.

Next, we investigated in detail *RP11-398K22.12* (ENSG00000229852), where the knockdowns by two independent ASOs (ASO_03, ASO_05) successfully reduced the expression of the target lncRNA (67%–82% knockdown efficiency, respectively) and further down-regulated its neighboring genes, *KCNQ5* and its divergent partner novel lncRNA *CATG00000088862.1* (Fig. 5A). Although the two genomic loci occupy Chromosome 6 and are 650 kb away, Hi-C analysis (Supplemental Methods; Supplemental Fig. S7; Supplemental Table S8) showed that they are located within the same topologically associated domain (TAD) and spatially colocalized (Fig. 5B). Moreover, chromatin-enrichment and single molecule RNA-FISH of *RP11-398K22.12* (Fig. 5C; Supplemental Table S9) suggested its highly localized *cis-*regulatory role.

In FANTOM5 (Hon et al. 2017), expression levels of *RP11-398K22.12*, *KCNQ5*, and *CATG00000088862.1* were enriched in brain and nervous system samples, whereas GTEx (The GTEx Consortium 2015) showed their highly specific expression in the brain, particularly in the cerebellum and the cerebellar hemisphere (Fig. 5D). GTEx data also showed that expression of *RP11-398K22.12* was highly correlated with the expression of *KCNQ5* and *CATG00000088862.1* across neuronal tissues (Fig. 5E,F), with the exception of cerebellum and the cerebellar hemisphere, potentially due to relatively lower levels of *KCNQ5* and *CATG00000088862.1*, whereas levels of *RP11-398K22.12* remained relatively higher. Additionally, we found an eQTL SNP (rs14526472) overlapping with *RP11-398K22.12* and regulating expression of *KCNQ5* in brain caudate (*P* = 4.2 × 10^−6^; normalized effect size −0.58). All these findings indicate that *RP11-398K22.12* is implicated in the nervous system by maintaining the expression of *KCNQ5* and *CATG00000088862.1* in a *cis-*acting manner.

## Discussion

This study systematically annotates lncRNAs through molecular and cellular phenotyping by selecting 285 lncRNAs from human dermal fibroblasts across a wide spectrum of expression, conservation levels and subcellular localization enrichments. Using ASO technology allowed observed phenotypes to be associated to the lncRNA transcripts, whereas, in contrast, CRISPR-based approaches may synchronically influence the transcription machinery at the site of the divergent promoter or affect regulatory elements of the targeted DNA site. Knockdown efficiencies obtained with ASOs were observed to be independent of lncRNA expression levels, subcellular localization, and of their genomic annotation, allowing us to apply the same knockdown technology to various classes of lncRNAs.

We investigated the *cis*-regulation of nearby divergent promoters, which has been reported as one of the functional roles of lncRNA (Luo et al. 2016). However, in agreement with previous studies (Guttman et al. 2011), we did not observe general patterns in the expression response of divergent promoters (Supplemental Fig. S3B). Recent studies suggest that transcription of lncRNA loci that do not overlap with other transcription units may influence RNA polymerase II occupancy on neighboring promoters and gene bodies (Engreitz et al. 2016a; Cho et al. 2018). Thus, it is plausible that transcription of targeted lncRNA was maintained, despite suppression of mature or nascent transcripts using ASOs. This further suggests that the functional responses described in this study are due to interference of processed transcripts present either in the nucleus, the cytoplasm, or both. Although it is arguable that ASOs may interfere with general transcription by targeting the 5′-end of nascent transcripts and thus releasing RNA polymerase II, followed by exonuclease-mediated decay and transcription termination (aka “torpedo model”) (Proudfoot 2016), most of the ASOs were designed across the entire length of the transcript. Since we did not broadly observe dysregulation in nearby genes, interference of transcription or splicing activity is less likely to occur.

We observed a reduction in cell growth for ∼7.7% of our target lncRNA genes, which is in line with previous experiments using CRISPRi-pooled screening, which reported 5.9% (in iPS cells) of lncRNAs exhibiting a cell growth phenotype (Liu et al. 2017). Although these rates are much lower than for protein-coding genes (Sokolova et al. 2017), recurrent observations of cell growth phenotypes (including cell death) strongly suggest that a substantial fraction of lncRNAs play an essential role in cellular physiology and viability. Further, when applying image-based analysis, we found that lncRNAs affect cell morphologies (Fig. 2G), which has not been so far thoroughly explored.

Several lncRNAs such as *MALAT1*, *NEAT1*, and *FIRRE* have been reported to orchestrate transcription, RNA processing, and gene expression (Kopp and Mendell 2018) but are not essential for mouse development or viability. These observations advocate for assays that can comprehensively profile the molecular changes inside perturbed cells. Therefore, in contrast to cell-based assays, functional elucidation via molecular phenotyping provides comprehensive information that cannot be captured by a single phenotypic assay. Herein, the number of overlapping differentially expressed genes between two ASOs of the same lncRNA targets indicated that 10.9% of lncRNAs exert a reproducible regulatory function in HDF.

Although the features of selected lncRNAs are generally similar to those of other lncRNAs expressed in HDFs (Fig. 1B–D), the cell-type-specific nature of lncRNAs and the relatively small sampling size (119 lncRNAs with knockdown transcriptome profiles) used in our study may not fully represent the whole extent of lncRNA in other cell types. However, lncRNA targets that did not exhibit a molecular phenotype may be biologically relevant in other cell types or cell states (Li and Chang 2014; Liu et al. 2017). At the same time, our results showed that particular lncRNAs expressed broadly in other tissues (e.g., in the human brain) were functional in HDFs (such as *RP11-398K22.12*). Although the exact molecular mechanisms of *RP11-398K22.*12 are not yet fully understood, its potential role in HDFs suggests that lncRNAs may be functionally relevant across multiple tissues in spite of the cell-type-specific expression of lncRNAs.

Further, we used siRNA technology to knockdown lncRNA targets as a method for independent validation. When comparing the transcriptomes perturbed by ASOs and siRNAs, concordance was observed only for three out of nine lncRNAs. This discrepancy is likely due to different modes of actions of the two technologies. Whereas ASOs invoke RNase H-mediated cleavage, primarily active in the nucleus, the siRNAs use the RNA-inducing silencing complex (RISC) mainly active in the cytoplasm. LncRNAs are known to function in specific subcellular compartments (Chen 2016) and their maturity, secondary structures, isoforms, and functions could be vastly different across compartments (Johnsson et al. 2013). Since the majority of functional lncRNAs are reported to be inside the nucleus (Palazzo and Lee 2018; Sun et al. 2018), ASO-mediated knockdowns, which mainly target nuclear RNAs, are generally more suitable for functional screenings of our lncRNA (62% found in the nuclear compartment). Besides, the dynamics of secondary effects mediated by different levels of knockdown from different technologies are likely to be observed as discordance when considering the whole transcriptome, where this kind of discordance has been reported previously (Stojic et al. 2018). In contrast, in the MKI67 assay, where only a single feature such as growth phenotype is assayed, siRNA knockdown revealed higher reproducibility with ASO knockdown. This suggested that the growth phenotype might be triggered by different specific pathways in ASO- and siRNA-knockdowns.

Previous studies suggest that lncRNAs regulate gene expression in *trans* epigenetically, via direct or indirect interaction with regulators such as DNMT1 (Di Ruscio et al. 2013) or by directly binding to DNA (triplex) (Mondal et al. 2015) or other RNA-binding proteins (Tichon et al. 2016). Analysis of cellular localization by fractionation followed by RNA-seq and in situ hybridization can indicate whether a given lncRNA may act in *trans* by quantifying its abundance in the nuclear soluble fraction as compared to cytoplasm. Although most lncRNAs in the nuclear soluble fraction may affect pathways associated with chromatin modification, additional experiments to globally understand their interaction partners will elucidate the molecular mechanism behind *trans*-acting lncRNAs (Li et al. 2017; Sridhar et al. 2017).

In summary, our study highlights the functional importance of lncRNAs regardless of their expression, localization, and conservation levels. Molecular phenotyping is a powerful and generally more sensitive to knockdown-mediated changes platform to reveal the functional relevance of lncRNAs that cannot be observed based on the cellular phenotypes alone. With additional molecular profiling techniques, such as RNA duplex maps in living cells to decode common structural motifs (Lu et al. 2016), and Oxford Nanopore Technology (ONT) to annotate the full-length variant isoforms of lncRNAs (Hardwick et al. 2019), the structure-to-functional relationship of lncRNAs may be elucidated further in the future.

## Methods

### Gene models and lncRNA target selections

The gene models used in this study were primarily based on the FANTOM CAGE-associated transcriptome (CAT) at permissive level as defined previously (Hon et al. 2017). From this merged assembly, there were ∼2000 lncRNAs robustly expressed in HDFs (TPM ≥ 1). However, we selected lncRNA knockdown targets in an unbiased manner to broadly cover various types of lncRNAs (TPM ≥ 0.2). Briefly, we first identified a list of the lncRNA genes expressed in HDFs, with RNA-seq expression at least 0.5 fragments per kilobase per million and CAGE expression at least 1 tag per million. Then, we manually inspected each lncRNA locus in the ZENBU genome browser for (1) its independence from neighboring genes on the same strand (if any), (2) support from RNA-seq (for exons and splicing junctions) and CAGE data (for TSSs) of its transcript models, and (3) support from histone marks at TSSs for transcription initiation (H3K27ac) and along the gene body for elongation (H3K36me3), from the Roadmap Epigenomics Consortium (Roadmap Epigenomics Consortium et al. 2015). A representative transcript model, which best represents the RNA-seq signal, was manually chosen from each locus for design of antisense oligonucleotides. In total, 285 lncRNA loci were chosen for ASO suppression. Additional controls (*NEAT1*, protein coding genes) (Supplemental Table S1) were added, including *MALAT1* as an experimental control. For details, please refer to the Supplemental Methods.

### ASO design

ASOs were designed as RNase H-recruiting locked nucleic acid (LNA) phosphorothioate gapmers with a central DNA gap flanked by 2–4 LNA nucleotides at the 5′ and 3′ ends of the ASOs. For details, please refer to the Supplemental Methods.

### Automated cell culturing, ASO transfection, and cell harvesting

Robotic automation (Hamilton) was established to provide a stable environment and accurate procedural timing control for cell culturing and transfection. In brief, trypsin-EDTA detachment, cell number and viability quantification, cell seeding, transfection, and cell harvesting were performed with automation. All transfections were divided into 28 runs on a weekly basis. ASO transfection was performed with duplication. In each run, there were 16 independent transfections with ASO negative control A (NC_A, Exiqon) and 16 wells transfected with an ASO targeting *MALAT-1* (Exiqon).

The HDF cells were seeded in 12-well plates with 80,000 cells in each well 24 h prior to the transfection. A final concentration of 20 nM ASO and 2 µL Lipofectamine RNAiMAX (Thermo Fisher Scientific) were mixed in 200 µL Opti-MEM (Thermo Fisher Scientific). The mixture was incubated at room temperature for 5 min and added to the cells, which were maintained in 1 mL complete medium. The cells were harvested 48 h posttransfection by adding 200 µL RLT buffer from the RNeasy 96 kit (Qiagen) after PBS washing. The harvested lysates were kept at −80°C. RNA was extracted from the lysate for real-time quantitative RT-PCR (Supplemental Methods).

### ASO transfection for real-time imaging

The HDF cells were transfected manually in 96-well plates to facilitate high-throughput real-time imaging. The cells were seeded 24 h before transfection at a density of 5200 cells per well. A final concentration of 20 nM ASO and 2 µL Lipofectamine RNAiMAX (Thermo Fisher Scientific) were mixed in 200 µL Opti-MEM (Thermo Fisher Scientific). After incubating at room temperature for 5 min, 18 µL of the transfection mix was added to 90 µL complete medium in each well. The ASOs were divided into 14 runs and transfected in duplicate. Each plate accommodated six wells of NC_A control, two wells of *MALAT1* ASO control, and two wells of mock-transfection (Lipofectamine alone) control.

Phase-contrast images of transfected cells were captured every 3 h for 2 d with three fields per well by the Incucyte live-cell imaging system (Essen Bioscience). The confluence in each field was analyzed by the Incucyte software. The mean confluence of each well was taken along the timeline until the mean confluence of the NC_A control in the same plate reached 90%. The growth rate in each well was calculated as the slope of a linear regression. A normalized growth rate of each replicate was calculated as the growth rate divided by the mean growth rate of the six NC_A controls from the same plate. Negative growth rate was derived when cells shrink and/or detach. As these rates of cell depletion could not be normalized by the rate of growth, negative values were maintained to indicate severe growth inhibition. Student's *t*-test was performed between the growth rate of the duplicated samples and the six NC_A controls, assuming equal variance.

### Cell morphology quantification

For each transfection, a representative phase-contrast image at a single time point was exported from the Incucyte time-series. These raw images were first transformed to probability maps of cells by pixel classification using ilastik (1.3.2) (Berg et al. 2019). The trained model was then applied to all images where the predicted probability maps of cells (grayscale, 16 bits tiff format) were subsequently used for morphology quantification in CellProfiler (3.1.5) (Carpenter et al. 2006). For details, please refer to the Supplemental Methods.

### MKI67 staining upon lncRNA knockdown

For the selected four lncRNA targets showing >25% growth inhibition, we used two siRNAs and two ASOs with independent sequences. The transfected cells were fixed by adding prechilled 70% ethanol and incubated at −20°C. The cells were washed with FACS buffer (2% FBS in PBS, 0.05% NaN3) twice. FITC-conjugated MKI67 (20Raj1, eBioscience) was applied to the cells and subjected to flow cytometric analysis. Knockdown efficiency by siRNA was determined by real-time quantitative RT-PCR using the same three primer pairs as for ASO knockdown efficiency. For details, please refer to the Supplemental Methods.

### Wound closure assay

The HDF cells were transfected with 20 nM ASO as described earlier in 12-well plates. The cells were replated at 24 h posttransfection into a 96-well ImageLock plate (Essen BioScience) at a density of 20,000 cells per well. At 24 h after seeding, cells form a spatially uniform monolayer with 95%–100% cell confluence. The cells were incubated with 5 µg/mL mitomycin C for 2 h to inhibit cell division. Then, medium was refreshed and a uniform scratch was created in each well by the WoundMaker (Essen BioScience). The closure of the wound was monitored by Incucyte live-cell imaging system (Essen Bioscience) every 2 h for 24 h. The RNA was harvested after the assay for real-time quantitative RT-PCR. For details, please refer to the Supplemental Methods.

#### Cap analysis of gene expression (CAGE)

Four micrograms of purified RNA were used to generate libraries according to the nAnT-iCAGE protocol (Murata et al. 2014). For details, please refer to the Supplemental Methods.

#### Chromosome conformation capture (Hi-C)

Hi-C libraries were prepared essentially as described previously (Lieberman-Aiden et al. 2009; Fraser et al. 2015a) with minor changes to improve the DNA yield of Hi-C products (Fraser et al. 2015b). For details, please refer to the Supplemental Methods.

## Data access

All raw and processed sequencing data generated in this study have been submitted to the DNA Data Bank of Japan (DDBJ; https://www.ddbj.nig.ac.jp/) under accession numbers DRA008311, DRA008312, DRA008436, and DRA008511 or can be accessed through the FANTOM6 project portal https://fantom.gsc.riken.jp/6/datafiles. The analysis results can be downloaded from https://fantom.gsc.riken.jp/6/suppl/Ramilowski_et_al_2020/data/ and interactively explored using our in-house portal https://fantom.gsc.riken.jp/zenbu/reports/#FANTOM6.

## Competing interest statement

The authors declare no competing interests.

## Supplementary Material

Supplemental Material
