# Supplementary material for: Functional annotation of human long noncoding RNAs via molecular phenotyping
Source: Genome Res. 2020 Jul;30(7):1060–72. doi: 10.1101/gr.254219.119 (PMC7397864; doi:10.1101/gr.254219.119)
Supplement: Supplemental Material [file supp_30_7_1060__index.html]

Functional annotation of human long noncoding RNAs via molecular phenotyping — Supplemental Material 

# Functional annotation of human long noncoding RNAs via molecular phenotyping

## Supplemental Material

- Supplemental\_Table\_S9.xlsx
- Supplemental\_Table\_S8.xlsx
- Supplemental\_Table\_S6.xlsx
- Supplemental\_Table\_S7.xlsx
- Supplemental\_Table\_S5.xlsx
- Supplemental\_Table\_S4.xlsx
- Supplemental\_Table\_S3.xlsx
- Supplemental\_Table\_S2.xlsx
- Supplemental\_Table\_S1.xlsx
- Supplemental\_Figures\_.pdf
- Supplemental\_Methods\_.docx
