# Supplementary material for: Functional annotation of human long noncoding RNAs via molecular phenotyping
Source: Genome Res. 2020 Jul;30(7):1060–72. doi: 10.1101/gr.254219.119 (PMC7397864; doi:10.1101/gr.254219.119)
Supplement: Supplemental Material [file supp_gr.254219.119_Supplemental_Figures_amended.pdf]

SUPPLEMENTAL FIGURES S1–7

Functional Annotation of Human Long Non-Coding RNAs via Molecular Phenotyping

Ramilowski Jordan A, Chi Wai Yip, *et al.*

Supplemental Figure S1. Efficacy of ASOs and correlation of knockdown efficiency with properties of lncRNAs and with cell growth and morphology changes.

Supplemental Figure S2. IncuCyte® growth inhibition and wound healing assessment.

Supplemental Figure S3. Transcriptional response of ASO knockdowns .

Supplemental Figure S4. Correlations of molecular phenotype with cell growth and morphologies.

Supplemental Figure S5. Transcriptional response of ASO and siRNA knockdowns (validations).

Supplemental Figure S6. Role of *ZNF213–AS1* in cancer.

Supplemental Figure S7. Hi-C protocol overview.

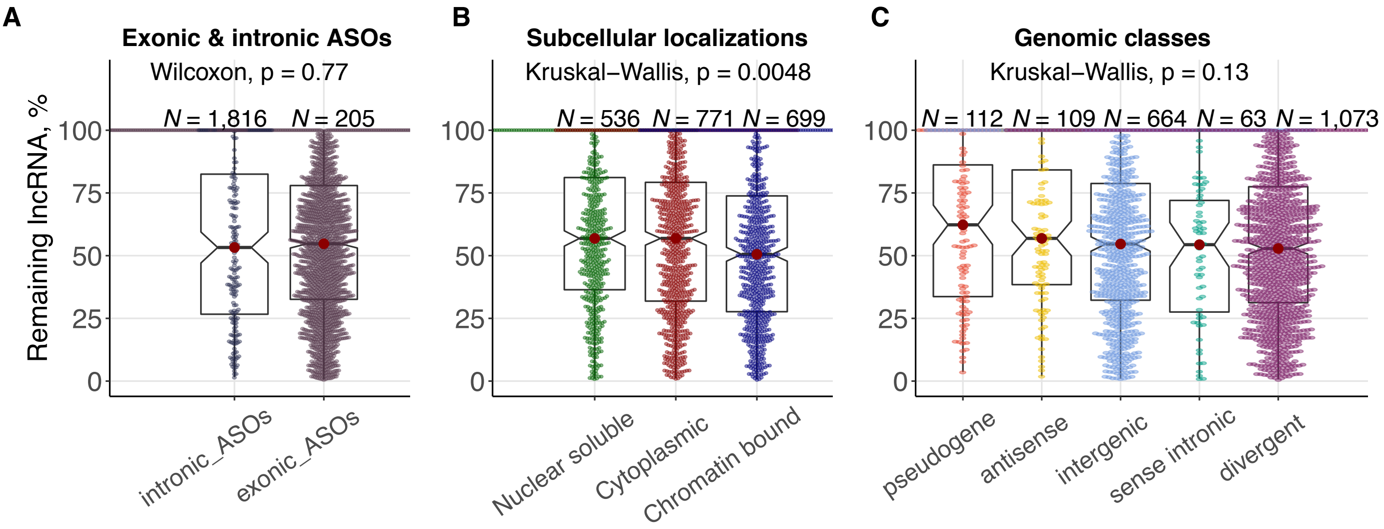

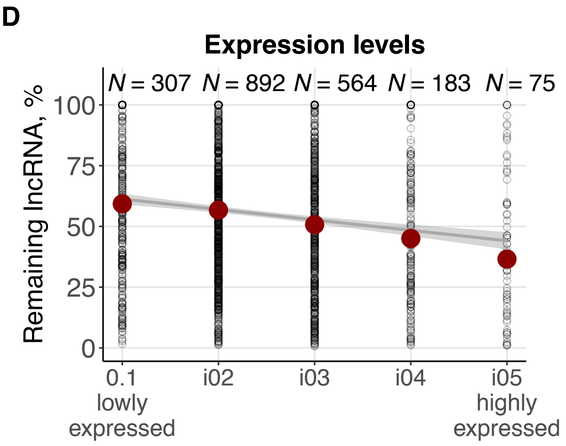

E

Cellular phenotype	Spearman (ρ)	P value
Extent	0.126	9.0×10^{-5}
Form Factor	0.150	4.3×10^{-6}
Solidity	0.114	0.00036
**Growth Rate**	**0.158**	**1.2×10^{-6}**
Area	0.161	8.2×10^{-7}
Compactness	0.145	8.2×10^{-6}
Eccentricity	0.166	3.9×10^{-7}
Perimeter	0.162	6.5×10^{-7}
Min Feret Diameter	0.182	2.7×10^{-8}
Max Feret Diameter	0.144	8.5×10^{-6}
Minor Axis Length	0.156	1.6×10^{-6}
Major Axis Length	0.140	1.5×10^{-5}
Max Radius	0.109	0.0006
Median Radius	0.075	0.013

Supplemental Figure S1. Efficacy of ASOs and correlation of knockdown efficiency with properties of lncRNAs and with cell growth and morphology changes. Comparison of KD efficiencies for: (*A*) ASOs targeting exonic and intronic regions of a given lncRNA, *(B)* lncRNA targets in different subcellular compartments, (*C*) different genomic classes of lncRNA targets and (*D*) different expression levels of lncRNA targets. (Highest KD efficiency across the three primer pairs used to measure each ASO knockdown is shown. Red dot indicates the median KD efficiency value. Negative KD efficiencies were set to 0). (*E*) Spearman correlations for KD efficiency and growth and cell morphology changes across 879 ASOs with successful KD (> 40% KD efficiency in at least two primer pairs or > 60% in one primer pair).

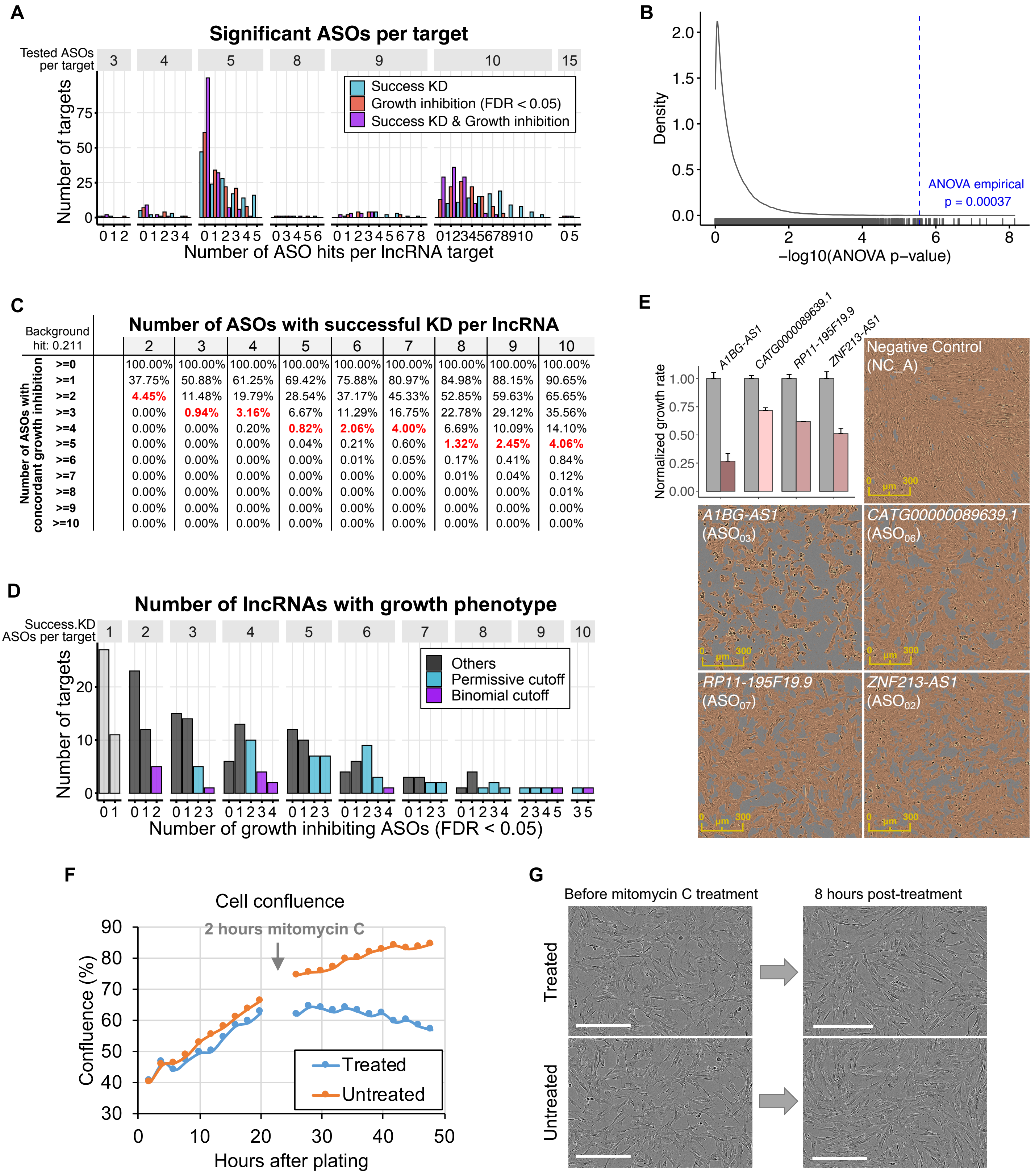

Supplemental Figure S2. **IncuCyte® growth inhibition and wound healing assessment**. (*A*) Number of growth inhibiting and successful knockdown (KD) ASOs per lncRNA target grouped by the number of tested ASOs per target. (*B*) Significance distribution (-log10(p)) for one-way ANOVA test for non-targeting ASOs (100,000 permutations) and the p value for targeting ASOs (blue line, p = 5.01 x 10^{-6}), which results in the empirical p value = 0.00037. (*C*) Growth inhibition binomial cutoffs depending on the number of ASOs with successful KD per target. (*D*) LncRNAs affecting cell growth with permissive cutoff (blue, >=2 ASO) and binomial cutoff (purple, cutoffs as in C) (*E*) Growth inhibition for selected lncRNA targets (grey bars indicate matched negative control samples) and the corresponding IncuCyte® images at 48 hours after transfection. (*F*) IncuCyte® cell confluence measurements across mitomycin C treatment time course. Treated cells were incubated with 5 µg/mL mitomycin C for 2 hours and then medium was removed and replaced with medium containing 0.5% FBS. Untreated controls were incubated in medium with 10% FBS throughout the time course. (*G*) Representative images taken before and 8 hours after adding mitomycin C showing similar morphologies and densities of the treated cells (upper panel) and untreated cells for comparison (lower panel). Scale bar: 300 µm.

A

Molecular phenotype	Spearman (ρ)	P value
DEGs	0.416	1.0×10^{-15}
GSEA	0.260	7.0×10^{-7}
MARA	0.174	0.00068

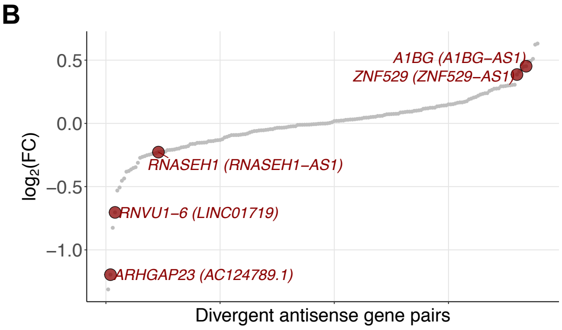

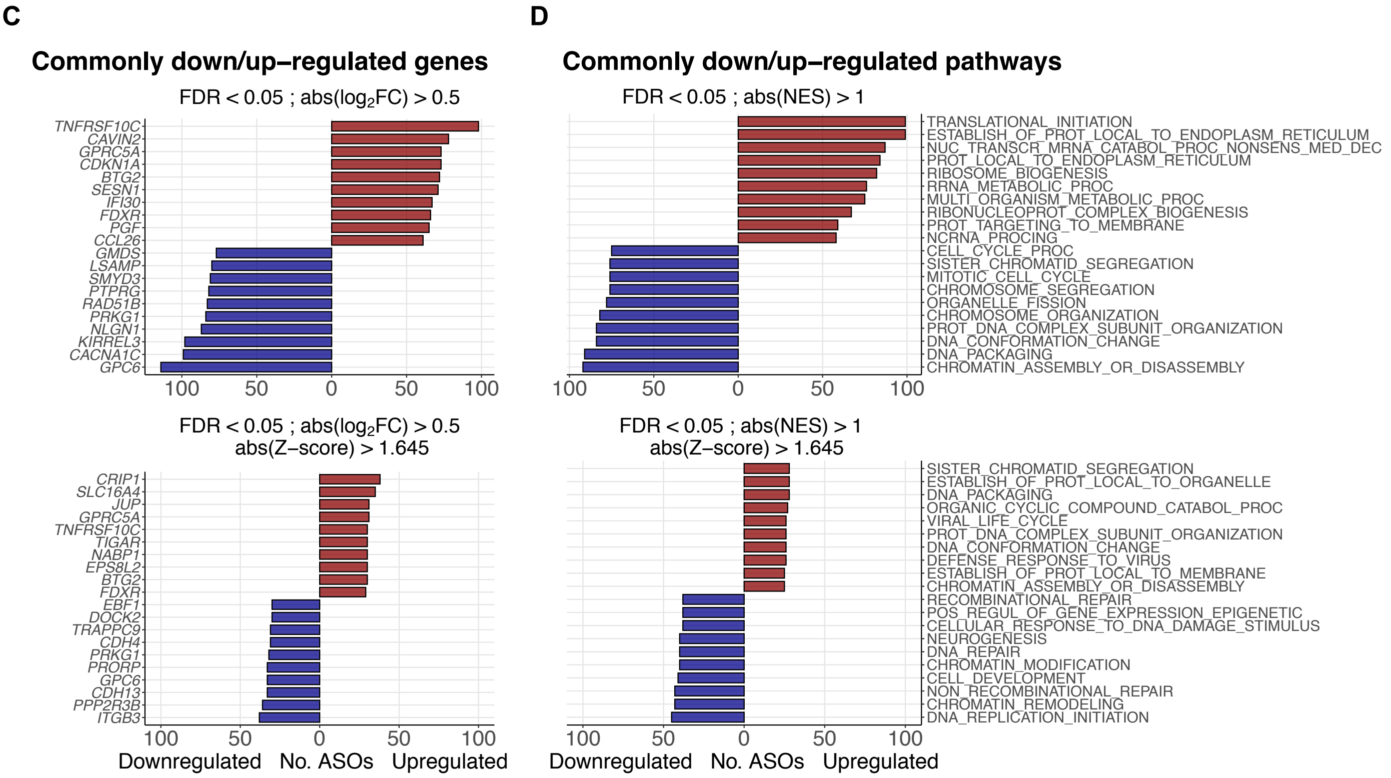

Supplemental Figure S3. Transcriptional response of ASO knockdowns (KDs). (*A*) Spearman correlations for KD efficiency and number of: DEGs, significantly changed GSEA pathways and MARA motifs across 335 ASOs (out of 340 ASOs) with KD > 0 (median KD efficiency ~ 71.9%). (*B*) Relative change in expression of the divergent-antisense to the lncRNA partner gene across 186 ASOs targeting 84 lncRNAs. *(C)* Top 10 down/up-regulated genes across 340 ASO KDs without the Z-score normalization (top) and with the Z-score normalization (bottom). (*D*) Same as in C, but for significantly enriched pathways.

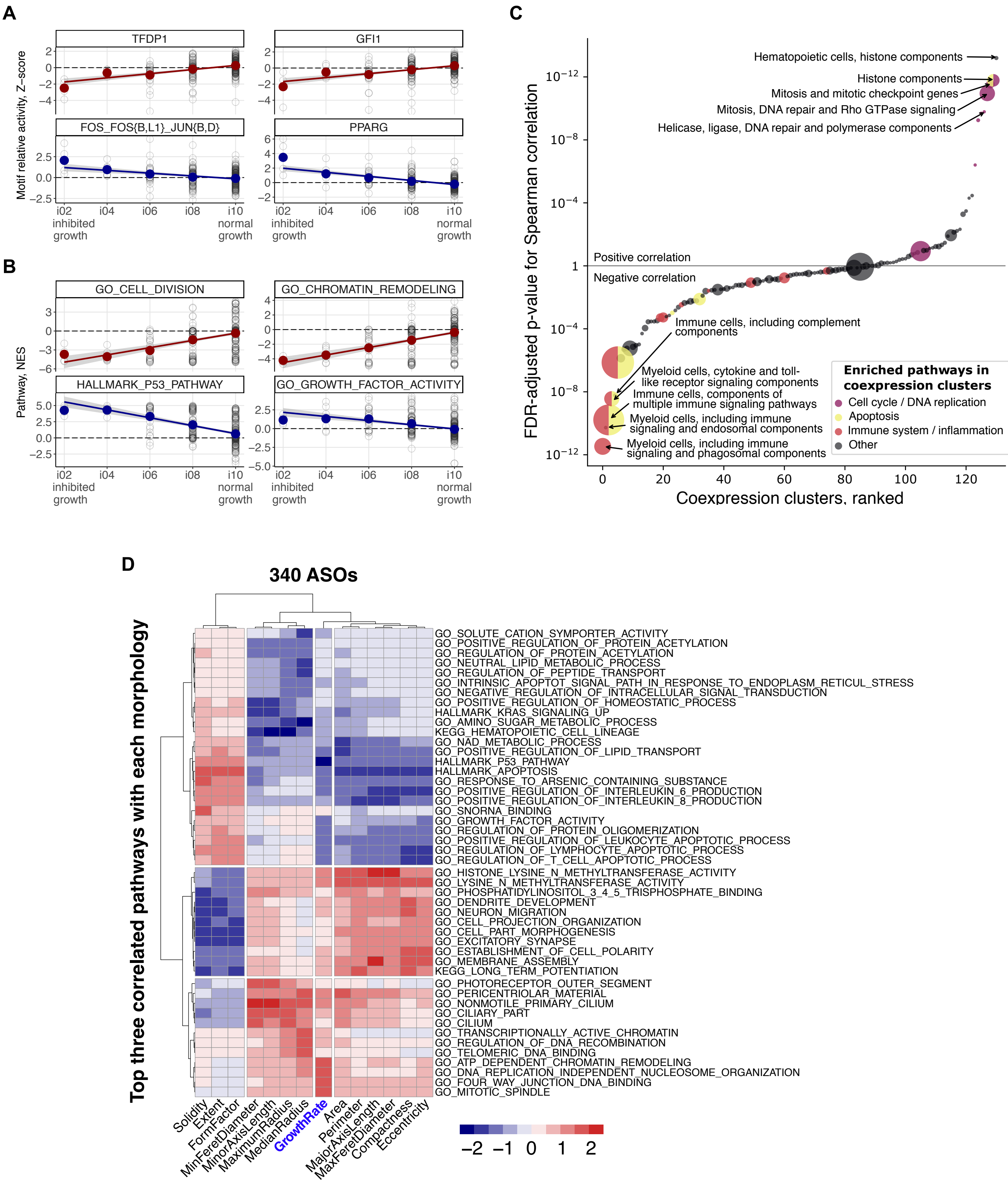

Supplemental Figure S4. **Correlations of molecular phenotype with cell growth and morphologies.** (*A*) Positive (red) and negative (blue) correlations for selected transcription factor binding motifs (relative motif activity) with the normalized growth rate across 340 ASOs targeting lncRNAs. Each hollow circle represents a single ASO. (*B*) Same as in A, but for selected GSEA pathways. (*C*) Global correlations of FANTOM 5 co-expression clusters enrichment with growth phenotype. (*D*) Top three significant GSEA pathways positively (red) and negatively (purple) correlated with the normalized growth rate and 13 cell morphologies (FDR-adjusted p value for Spearman correlation re-scaled across morphologies).

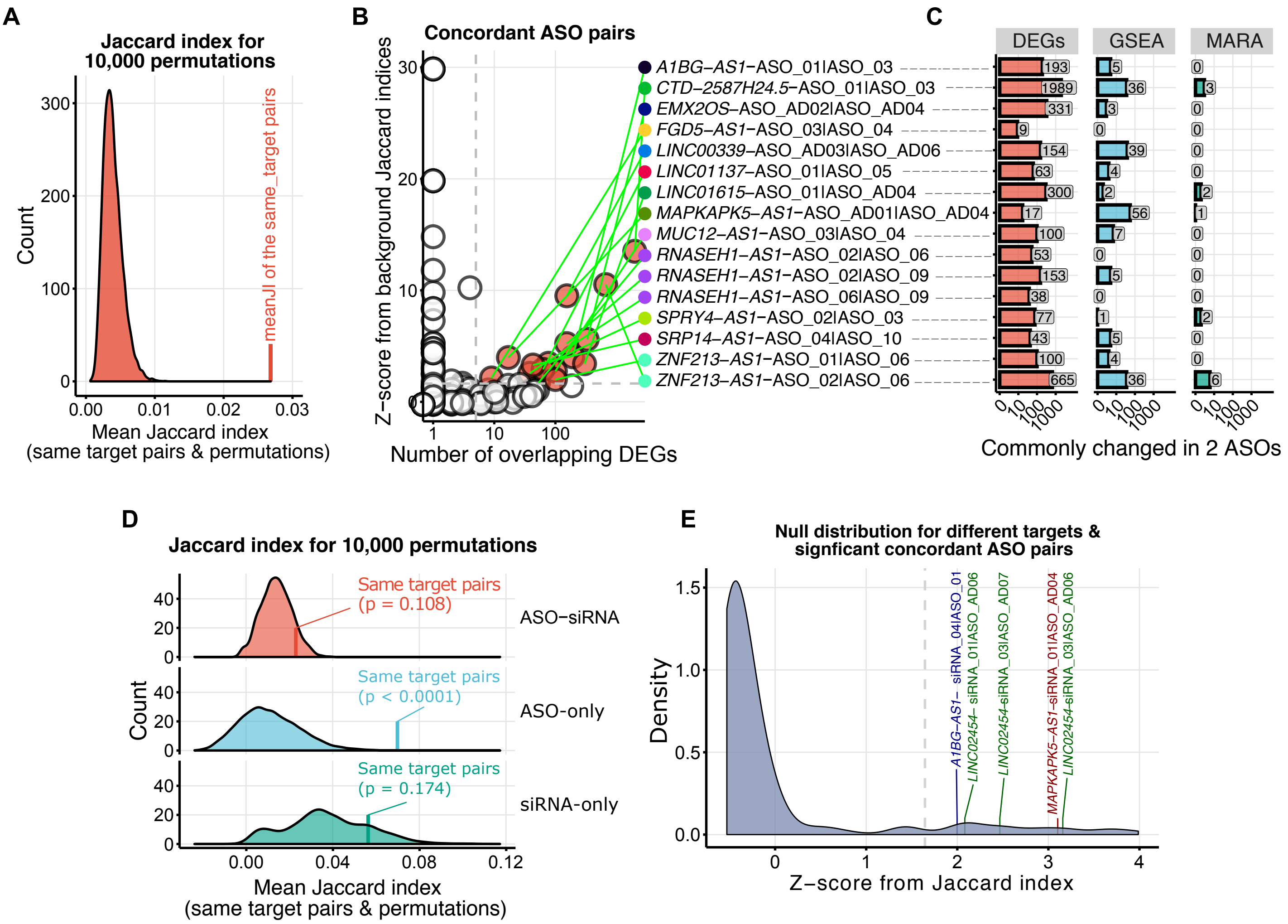

Supplemental Figure S5. Transcriptional response of ASO and siRNA knockdowns (validations). (*A*) Jaccard indices across 10,000 permutations for the concordance of the same target group with all possible pairs ($N = 277$). Permutations were performed by shuffling one side of the pairs while enforcing the two ASOs from each pair to target different genes. (*B*) 16 ASO pairs targeting 13/119 (10.9%) of lncRNAs exhibiting reproducible molecular response based on their Jaccard indices significantly above the background ($p < 0.05$) and with at least five common DEGs (FDR < 0.05, abs(log2FC) > 0.5, abs(Z-score) > 1.645). (*C*) Numbers of overlapping DEGs, GSEA pathways and MARA motifs for the 16 ASO pairs described in (B). (*D*) Jaccard index for common DEGs (FDR < 0.05, abs(log2FC) > 2) for ASO–ASO, ASO–siRNA and siRNA–siRNA pairs. The procedures is described in (A). (*E*) Concordant response for (5/36) ASO-siRNA pairs targeting three lncRNAs, based on their Jaccard indices significantly above the background ($p < 0.05$) and a requirement of at least five common DEGs.

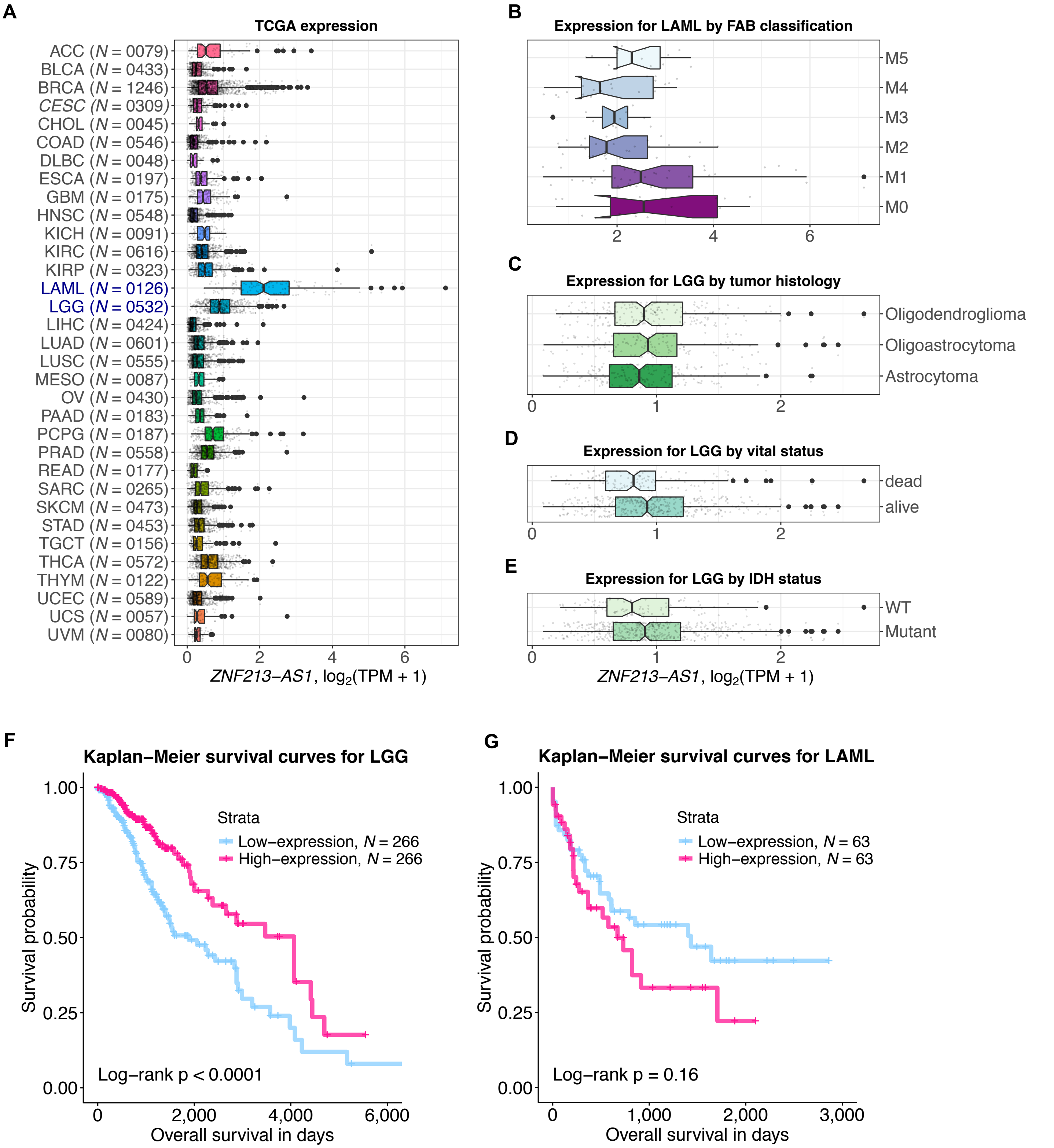

Supplemental Figure S6. Role of *ZNF213–AS1* in cancer. (*A*) Expression of *ZNF213-AS1* in TCGA. (*B*) Expression of *ZNF213-AS1* for Acute Myeloid Leukemia (LAML) by French-American-British classification. The levels are higher in M0 and M1 subtypes. These subtypes are considered the most undifferentiated stages of LAML. Since *ZNF213-AS1* shows the lowest expression level in blood cells (GTEx), the higher level of expression could be suggestive of dysregulation of its expression in undifferentiated stages or involvement in early precursor of blood cells. (*C*) Expression of *ZNF213-AS1* in Low Grade Glioma (LGG) by tumor histology classification. Oligodendrocyte differentiation is related to good prognosis is this cancer type. (*D*) Expression of *ZNF213-AS1* in LGG by tumor IDH (isocitrate dehydrogenase) mutational status. A total of 511 samples with mutational information were available, 417 of them were classified as "Mutant" and 94 as "wild type". IDH mutation has been repeatedly associated with better prognosis than wild type in gliomas. (*E*) Expression of *ZNF213-AS1* by patients' vital status. The number of alive patients is 387 out of the total of 513 with available follow-up. Univariate Cox proportional hazard analysis as well as Kaplan-Meier curves for: (*F*) LGG (HR: 0.61, FDR: 0.0079), (*G*) LAML (HR: 1.32; FDR: 0.5455).

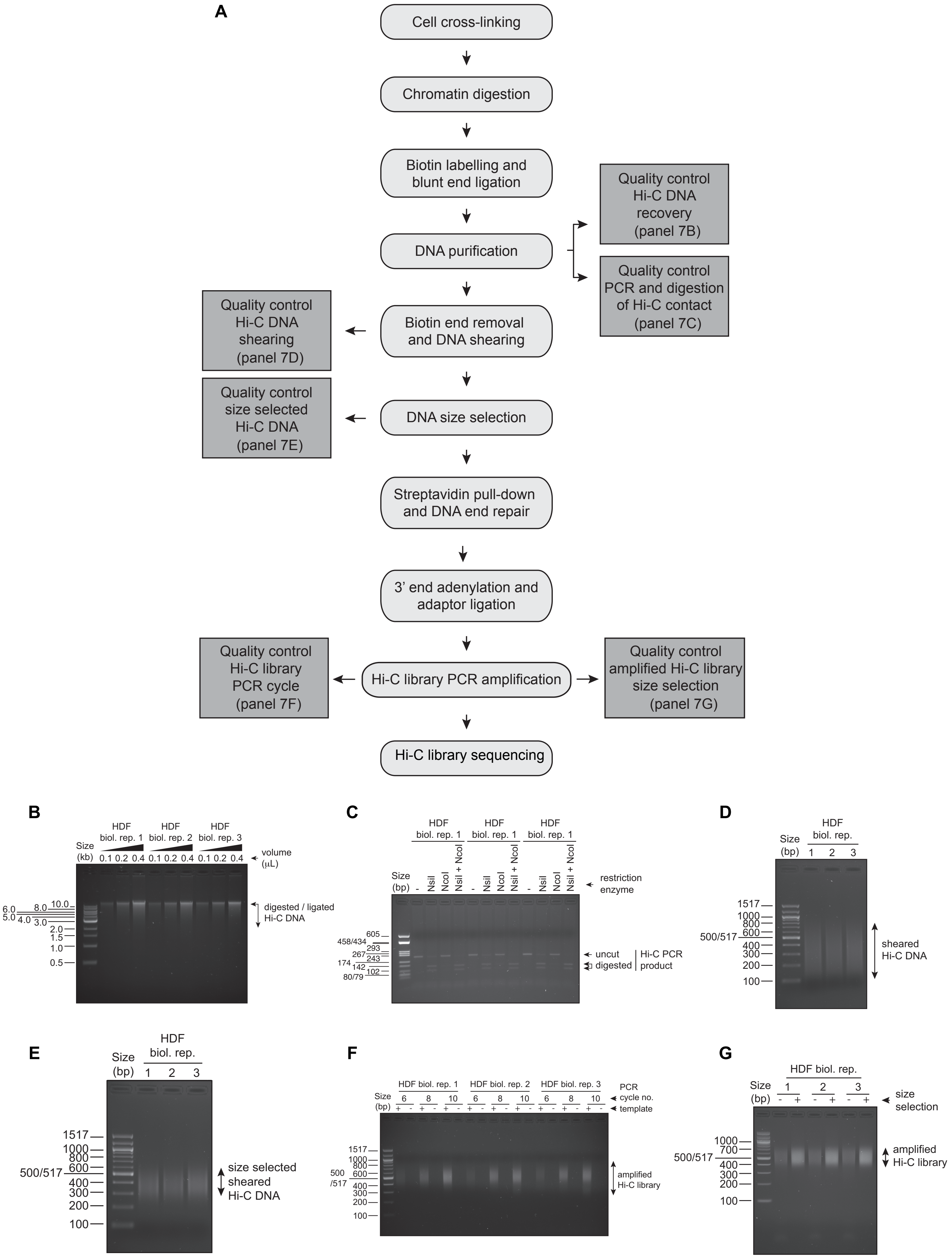

Supplemental Figure S7. Hi-C protocol overview. (*A*) Flowchart of the Hi-C protocol with related quality control (QC) steps: (*B*) cDNA recovery quality control, (*C*) PCR and digestion QC, (*D*) DNA shearing QC, (*E*) DNA size-selection QC, (*F*) PCR cycle QC and (*G*) DNA size-selection QC.
